# Nesting Biology and Fungiculture of the Fungus-Growing Ant, *Mycetagroicus cerradensis*: New Light on the Origin of Higher Attine Agriculture

**DOI:** 10.1673/031.011.0112

**Published:** 2011-02-04

**Authors:** Scott E. Solomon, Cauê T. Lopes, Ulrich G. Mueller, Andre Rodrigues, Jeffrey Sosa-Calvo, Ted R. Schultz, Heraldo L. Vasconcelos

**Affiliations:** ^1^ Department of Entomology, National Museum of Natural History, Smithsonian Institution, POB 37012, Washington, DC 20013-7012, U.S.A.; ^2^ Institute of Biology, Federal University of Uberlândia (UFU), C.P. 593, Uberlândia, MG, 38400–902, Brazil; ^3^ Section of Integrative Biology, The University of Texas at Austin, 1 University Station C0930, Austin, TX, 78712, U.S.A.; ^4^ Center for the Study of Social Insects (CEIS), State University of São Paulo, Av. 24-A 1515, Rio Claro, SP, 13506- 900, Brazil; ^5^ Maryland Center for Systematic Entomology, Department of Entomology, University of Maryland, 4112 Plant Sciences Building, College Park, MD, 20742, U.S.A; ^6^ Current address: Department of Ecology and Evolutionary Biology, Rice University, 6100 Main Street, MS 170, Houston, TX 77005, U.S.A.

**Keywords:** Attini, Cerrado, evolutionary transitions, *Leucocoprinus*, molecular systematics, nest architecture

## Abstract

The genus *Mycetagroicus* is perhaps the least known of all fungus-growing ant genera, having been first described in 2001 from museum specimens. A recent molecular phylogenetic analysis of the fungus-growing ants demonstrated that *Mycetagroicus* is the sister to all higher attine ants (*Trachymyrmex, Sericomyrmex, Acromyrmex, Pseudoatta*, and *Atta*), making it of extreme importance for understanding the transition between lower and higher attine agriculture. Four nests of *Mycetagroicus cerradensis* near Uberlândia, Minas Gerais, Brazil were excavated, and fungus chambers for one were located at a depth of 3.5 meters. Based on its lack of gongylidia (hyphal-tip swellings typical of higher attine cultivars), and a phylogenetic analysis of the ITS rDNA gene region, *M. cerradensis* cultivates a lower attine fungus in Clade 2 of lower attine (G3) fungi. This finding refines a previous estimate for the origin of higher attine agriculture, an event that can now be dated at approximately 21–25 mya in the ancestor of extant species of *Trachymyrmex* and *Sericomyrmex*.

## Introduction

Species of the tribe Attini (Formicidae, Myrmicinae) are unique among the ants in their obligate association with fungi that are cultivated for food (Weber 1972). The attines, or fungus-growing ants, comprise more than 230 described species that range from the United States to Argentina with a center of diversity in the Neotropics (Bolton et al. 2006; Hölldobler and Wilson 1990). The most common form of fungal agriculture practiced by extant attine species (“lower agriculture”) is also the most ancient (Mueller et al. 1998): it is thought to have originated approximately 50 million years ago (Schultz and Brady 2008) and subsequently to have given rise to four other distinct agricultural systems. Of these, the most celebrated—and problematic for human agriculture—is known as “higher” agriculture.

**Figure 1.**
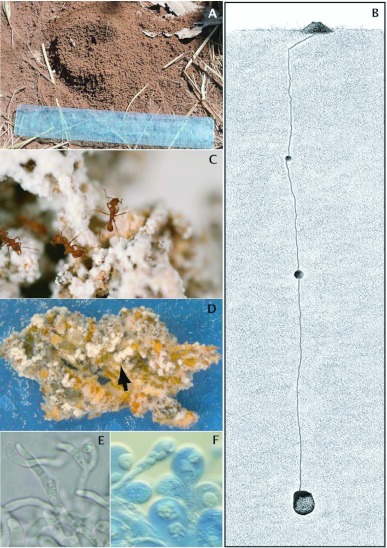
Nest and fungus garden of Mycetagroicus cerradensis. (A) Entrance of nest TRS080923-01 in cerrado sensu stricto (ruler is 15 cm long). (B) Sketch of cross section through a nest of Mycetagroicus cerradensis, showing the approximate relative locations of the entrance mound, tunnels, empty chambers, and fungus garden chamber (note that this drawing is not to scale; actual depth to top of fungus garden chamber is 354 cm). (C) Mycetagroicus cerradensis workers tending their fungal garden. (D) A piece offungal garden several days after excavation (arrow shows white mycelial swellings; see text for details). (E) Mycetagroicus cerradensis fungal cultivar hyphae under 100x magnification. (F) Cultivar of Atta cephalotes (stained with cotton-blue) with swollen hyphae (gongylidia) typical of higher attine (“G1”) cultivars. Photo credits: A, C by S. Solomon; D, E by A. Rodrigues; F by U. Mueller. Drawing B by Karolyn Darrow. High quality figures are available online.

Higher attine agriculture, which recent data suggest originated between 15 and 26 million years ago (Schultz and Brady 2008), is practiced by four extant genera of attine ants: the leafcutter genera *Acromyrmex* and *Atta*, and the non-leafcutters *Sericomyrmex* and *Trachymyrmex*. Higher attine fungi are distinct from all other fungi cultivated by attines because they produce hyphal swellings called “gongylidia” grown in clusters called staphylae that contain a high concentration of nutrients. Staphylae are visible to the naked eye and can be easily recognized under a microscope (Figure 1F). They are presumably produced by the fungi for the benefit of their ant hosts (Weber 1972).

Higher attine fungi are also distinct in that, unlike lower attine fungi, they are not thought to be capable of living outside the ants' nests (Mueller 2002). However, molecular analyses suggest that higher attine cultivars are capable of sexual reproduction and likely disperse as spores across long distances (Mikheyev et al. 2006; Mikheyev et al. 2007). Indeed, whereas mushrooms sprouting out of leafcutter ant gardens have been observed a number of times (reviewed in Mueller 2002), no fruiting bodies of leafcutter fungi have yet been found unassociated with a leafcutter ant nest (reviewed in Vo et al. 2009). In contrast, numerous fruiting structures closely related to those cultivated by lower attine ants — some identical in fast-evolving DNA sequences to those of cultivated strains—have been found growing in the wild, without any obvious association with attine nests (Mueller et al. 1998; Vo et al. 2009). This observation, along with the observation that sequence-identical fungi are found in distantly-related ant nests (Green et al. 2002; Mueller et al. 2004), have led to the conclusion that ants participating in lower attine agriculture regularly recruit new fungi from wild stocks, or that fungi associated with lower attine ants regularly “escape” from the ants' nests, or both (Mueller et al. 1998).

The evolutionary transition from lower to higher attine agriculture (and subsequently to leafcutter agriculture) was a key innovation that resulted in an adaptive explosion of ant species beginning around 20 million years ago. Several biological and life-history characteristics accompanied this transition, including changes in colony size, worker size, worker/worker and worker/queen polymorphism, foraging substrate, queen mating frequency, mode of nest founding, and susceptibility to infection by the parasite *Escovopsis* (Currie 2001; Schultz and Brady 2008; Villesen et al. 2002). However, the closest living descendents of the lineages that participated in this important event are among the most enigmatic of all attines, rendering difficult any effort to elucidate the evolutionary sequence of events. Of particular relevance here are the four species of the recently-described genus *Mycetagroicus* Brandão and Mayhé-Nunes (Brandão and Mayhé-Nunes 2001), which occupy a phylogenetic position intermediate between the lower attines (*Cyphomyrmex wheeleri* group) and the higher attines (Schultz and Brady 2008). Because the biology of *Mycetagroicus* is virtually unknown, and specifically because it is not known whether these species practice higher or lower agriculture, it is currently unclear what ancestral species was the first to domesticate fungi: the common ancestor of (*Mycetagroicus* + the higher attines) or the common ancestor of the two most basally diverging lineages within the higher attines, consisting of the *Trachymyrmex* *urichi/opulentus* group + *Sericomyrmex* and all other *Trachymyrmex* + *Acromyrmex* + *Atta*.

In order to determine whether *Mycetagroicus* ants participate in higher or lower attine agriculture as well as to better understand the natural history of this little-known genus nests of *Mycetagroicus cerradensis* near Uberlândia, Minas Gerais, Brazil were excavated. Observations were made on the nest architecture and behavior of this species in the field, live ants and fungi were collected for microbial isolations and laboratory observations, and fungal DNA was sequenced to determine its phylogenetic position among attine fungal cultivar strains.

## Materials and Methods

### Field observations and nest excavations

Fieldwork was carried out from 20–26 September 2008 at Estação Ecológica do Panga (19.17291° S, 48.3967° W, elevation 813 m), in a 404 ha reserve located 30 km south of Uberlândia, Minas Gerais, Brazil. The region is characterized by a subtropical climate with two well-defined seasons: a dry winter (May to September) and a rainy summer (October to April). The mean annual temperature and precipitation are 22° C and 1650 mm, respectively. Soils at the site are primarily red latosols. The reserve is covered by a mosaic of vegetation types typical of the *Cerrado* biome (Oliveira-Filho and Ratter 2002), including savannas with a sparse tree cover (*campo cerrado*), savannas with 30–60% of tree cover (*cerrado sensu stricto*), and forests (*cerradão* and semideciduous forest).

Nests were located by baiting workers and following them to the nest entrances. Four different nests were located; two were in the transition zone between *cerradão* and *cerrado sensu stricto*, one in the transition zone between *cerrado sensu stricto* and *campo cerrado* and one in *campo cerrado*. Excavations were conducted by first digging a large trench at a distance of approximately half a meter from the nest entrance, but without disturbing the nest proper. The ants' tunnel was then traced by inserting a blade of grass or other flexible material into the tunnel and carefully scraping away the surrounding dirt so that the original tunnel structure remained visible. Measurements and photographs of all tunnels and chambers were taken to document nest architecture. Once a fungus chamber was found, all worker ants seen were collected either live into plastic containers with moistened plaster bottoms previously sterilized under UV light, or preserved in vials containing 95% ethanol. Nest fragments containing fungi and ants that did not come into contact with soil were collected using sterilized forceps and served as the material for subsequent microbial isolations (see below).

### *Escovopsis* screens

After collection, the ants were allowed to rebuild their fungus garden for two days in a UV-sterilized container before microbial isolations began. Fifteen garden pieces (about 3 mm3) were removed using sterile forceps and 5 garden pieces per plate were plated onto potato-dextrose agar (PDA) medium supplemented with 50 μg/ml of penicillin G and streptomycin (Sigma). Plates were incubated in the dark at 25° C for seven days and were monitored daily for fungal growth. A second isolation round (15 additional fragments) was performed five days after the first attempt was begun.

## Molecular analyses

A small quantity of cultivar mycelium was teased from the ethanol-preserved garden material with sterilized forceps and Chelexextracted following the methods of Sen et al. (2010). Amplification and sequencing of the ITS region followed the methods of Mueller et al. (1998). Both forward and reverse ITSsequences were generated and then compiled in a contig-sequence (deposited under accession HM245775 at GenBank).

### Phylogenetic analyses

**Figure 2.**
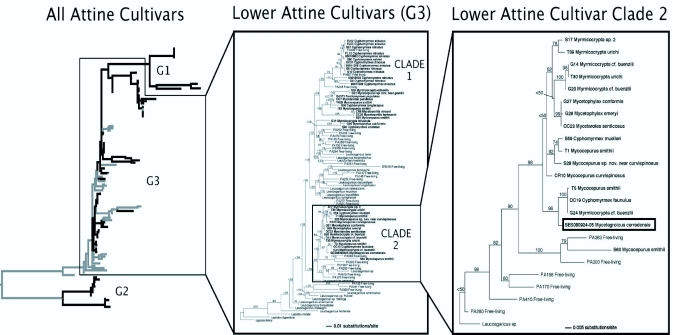
Phylogenetic placement of fungi cultivated by *Mycetagroicus cerradensis*. **Left:** Overview of phylogenetic relationships among fungi cultivated by attine ants, with fungal lineages associated with ants depicted in black and free-living fungal lineages in grey (adapted from Mikheyev et al. 2010). **Middle:** (“Global analysis”) Phylogeny of lower-attine cultivars (“G3” sensu Chapela et al. 1994) and closely related free-living fungi reconstructed using the internal transcribed spacer (ITS) rDNA region. The most likely tree with support values from 100 bootstrap replicates above each node is shown. Fungi cultivated by attine ant species are shown in bold (indicated by name of ant host) and free-living fungi in normal font. Lower-attine Clade 1 and Clade 2 cultivars are named as in Mueller et al. (1998). **Right:** (“Focal analysis”) Phylogeny of Clade 2 lower-attine cultivars and closely related free-living fungi using the ITS region. Shown here is the most likely tree with support values from 1000 bootstrap replicates. Fungi cultivated by attine ant species are shown in bold (indicated by name of ant host) and free-living fungi in normal font. Fungus cultivated by *Mycetagroicus cerradensis* is outlined with a black box. High quality figures are available online.

The single DNA sequence of the ITS region was added to a reduced-taxon version of the alignment of lower attine cultivars used by Vo et al. (2009) and aligned by hand using MacClade 4.06 (Maddison and Maddison 2000). An initial (“global”) phylogenetic analysis consisting of 601 basepairs (after the removal of unalignable positions) and 92 taxa spanning cultivar Clades 1 and 2, as well as closely related free-living fungi, was conducted to determine the broad placement of the *Mycetagroicus* cultivar with respect to other attine cultivars. Using the results of this initial analysis, a second alignment, consisting of only Clade 2 cultivars and closely related free-living fungi (24 taxa, 720 basepairs), was used to determine a more precise (“focal”) placement for the *Mycetagroicus* cultivar. The difference in the lengths of the two alignments is due to several sections of the ITS region that could not be unambiguously aligned in the global analysis, and these unalignable regions were therefore excluded from the global phylogenetic analyses. Maximum likelihood searches were conducted for both alignments in Garli 0.951 (Zwickl 2006) using default settings and 100 (for global analysis; Figure 2 middle panel), or 1000 (for focal analysis; Figure 2 right panel) bootstrap replicates. A consensus tree summarizing the bootstrap analyses was constructed using PAUP* 4.0b10 (Swofford 2003).

## Results

### Nest locations and architecture

Four nests of *Mycetagroicus cerradensis* were located at Estação Ecológica do Panga. Excavations were attempted from 23–26 September 2008 for all four nests, although in only one case (SES080924-05) were chambers found containing fungus garden. All four nests displayed similar nest architecture in the excavated portions of the nests (Figure 1B).

The entrances of all four nests contained a single entrance hole. In three of the four nests (UGM080923-01, TRS080923-01, CTL080924-01), the entrance was surrounded by a mound of excavated soil of varying size. The nest that did not have a mound was located on a trail, and any above-ground structure was almost certainly destroyed by foot travel before the nest was noticed. Measurements of the nest entrance were made only for nest TRS080923-01, which had a mound made of coarse, reddish-brown soil pellets that was 11 cm by 9 cm at its base and 3 cm high, with a circular crater that was 5 cm by 4 cm (Figure 1A). The entrance hole at the base of the crater was 5 mm in diameter.

In all four nests, the entrance hole led to a tunnel that proceeded downward from the surface of the ground at an angle that departed slightly from the vertical so that the first chamber was not located directly beneath the nest entrance. This small chamber, which did not contain any fungus garden, was located at a depth of 2–10 cm beneath the surface. In two nests (UGM080923-01 and SES080924-05), this chamber marked the location where the tunnel began to descend vertically (i.e. perpendicular to the surface of the ground). In the case of nest TRS080923-01, the point at which the tunnel turned and began to descend vertically was not directly observed because this section of the nest was destroyed during the excavation; however the turn (located approximately 28 cm below the surface and 20 cm measured horizontally from the nest entrance) was inferred from the two tunnel segments that were observed on either side of it.

In the three cases in which the excavations were able to follow the vertical tunnel (it could not be located in CTL080924-01), this shaft, which measured 3–4 mm in diameter, led downwards for 143–371 cm before reaching another chamber (although a slight swelling of approximately 1.5 cm was located at a depth of 83 cm in TRS080923-01). At these depths, two of the nests (UGM08092301 and TRS080923-01) had chambers filled loosely with soil. In UGM080923-01, the vertical shaft led to a single soil-filled chamber at a depth of 216 cm. The chamber was 10 cm high, 5 cm wide, and 11 cm deep. Despite careful excavation around and below this chamber, no additional chambers were found. However, a horizontal tunnel was discovered at the approximate depth of the chamber, though it was largely destroyed during the excavation. The excavation was left open overnight and no signs of worker activity or excavation were observed the following day, suggesting that either no other chambers existed, or that the colony was sufficiently disturbed that all activity had ceased.

In nest TRS080923-01 the vertical shaft led to a spherical chamber, roughly 3 cm in height and width, located 143 cm below the surface that was filled with loose soil. Below this chamber, the shaft continued to descend straight down to a second chamber, also mostly filled with loose soil at a depth of 160 cm below the surface. This chamber was irregularly shaped, with a main compartment that had a height of 8 cm and width of 3 cm. Within this chamber, a pocket in the back that was 2 cm high extended the width of the chamber at least another 7 cm. Additionally, a second tunnel (distinct from the vertical shaft that connected to the top of this chamber) connected to this chamber near the top of the back wall (as seen by the excavator). This tunnel led back, away from the plane of excavation, and curved until the tunnel descended vertically at a slight angle from the plane of excavation, for approximately 34 cm. Three additional chambers were located at approximately 260 cm beneath the surface, all filled with loose soil and measuring 4 cm high by 5 cm wide, 4 cm high by 4 cm wide, and 7 cm high by 7 cm wide. No connections between these three chambers and the vertical tunnel were found. Excavation of TRS080923-01 continued to a depth of 290 cm and failed to locate a garden-containing chamber.

Nest SES080924-05 contained two chambers with fungus gardens. The first chamber was located at a depth of 354 cm beneath the ground surface, and measured approximately 5 cm high by 9 cm wide (exact measurements were not possible because the chamber was partially destroyed during the excavation). This chamber was located 17 cm away from the vertical shaft, but the tunnel that presumably connected it to the shaft was not located. A second chamber containing fungus garden was located at the terminus of the vertical shaft, 371 cm beneath the surface. This chamber was 4 cm high and 10 cm wide. Both chambers were completely filled with fungus garden (Figure 1C), which appeared to be sessile, although small rootlets, which may have suspended part of the garden, did pass through the chambers and were in contact with parts of the garden. No additional tunnels or chambers were found, despite careful excavation around (10–15 cm laterally) and below these chambers to a depth of 390 cm, and less careful excavation to a depth of 410 cm.

Although no tunnel was found connecting one of the garden-containing chambers with the other chamber or with the vertical shaft, three lines of evidence suggest that these chambers were part of the same colony: (1) they were located only 17 cm apart, and a survey of attine nests at this site (Vasconcelos et al. 2008) suggests that *Mycetagroicus* nests do not occur at high nest densities; (2) only a single queen was found between the two chambers despite careful attempts to collect all individuals; and (3) when worker ants from both chambers were placed together in a single container no signs of aggression were observed.

At the time of excavation, 302 worker ants were collected as vouchers from nest SES080924-05. In addition to the ants, several nitidulid beetles were found inside the nest chambers and tunnels of this nest and nest TRS080923-01. In an attempt to rear worker-produced males (males are currently undescribed from *M. cerradensis*), two fragments of this nest were kept alive in the lab for several months. Both fragments contained fungus garden, worker ants, and brood, and one fragment contained the queen. The queen died in February 2009, at which time she and all 350 remaining workers from that colony fragment were preserved in 95% ethanol. The queenless fragment failed to produce males by 27 March 2009, at which time the entire colony (94 workers and the entire fungus garden) was preserved in 95% ethanol. Vouchers for all material will be deposited in the entomology collections at the USNM (Washington, DC, USA), the UFU (Uberlândia, MG, Brazil), and MZSP (São Paulo, SP, Brazil).

### Fungiculture

The fungus garden had small, whitish nodules on its external surface as well as in its interior (Figure 1D). Examination under a microscope (Figure 1E) revealed that these structures are not the clusters of gongylidia typical of higher attine (G1) cultivars (Figure 1F), but instead resemble the swollen hyphae often seen in lower attine Clade-2 cultivars, such as those cultivated by some species of *Cyphomyrmex* and *Myrmicocrypta*. Indeed, phylogenetic analyses of the ITS region of the fungal cultivar (Figure 2) indicate that it falls within Clade 2 of the lower attine (G3) cultivars (Chapela et al. 1994; Mueller et al. 1998), in a subgroup of fungi cultivated by *Cyphomyrmex faunulus, Mycocepurus smithii*, and *Myrmicocrypta* cf. *buenzlii* (Figure 2). This position is well supported by maximum likelihood bootstrap support values of 97% in the global analysis (Clades 1 and 2 plus closely related free-living fungi; Figure 2, middle panel) and 96% in the focal analysis (only Clade 2 and close relatives; Figure 2, right panel).

In isolations of filamentous fungi from the *Mycetagroicus* garden, *Escovopsis* was not detected in any of the 30 garden fragments plated onto PDA medium, although other filamentous fungi, such as *Trichoderma* spp., were present.

## Discussion

This study is the first to describe a nest and fungus garden of any species of *Mycetagroicus*. Based on our observations and molecular phylogenetic analyses of the fungi cultivated by one nest of *Mycetagroicus cerradensis*, this species cultivates fungi in Clade 2 of the lower attine (G3) cultivar group (Mueller et al. 1998) (Figure 2). Although a recent molecular phylogenetic reconstruction of the attine ants (Schultz and Brady 2008) suggested that *Mycetagroicus* is the sister group to the higher attine ants, supporting the conjecture by Brandão and Mayhé-Nunes (2008) that *Mycetagroicus* is a higher attine, our data indicate that, at least with regard to fungiculture, the genus *Mycetagroicus* belongs to the lower attines.

The nest architecture of *Mycetagroicus cerradensis* (Figure 1B) is roughly similar to that of some other lower attines (Diehl-Fleig and Diehl 2007; Fernández-Marín et al. 2005; Klingenberg and Brandão 2009; Klingenberg et al. 2007; Mueller and Wcislo 1998; Rabeling et al. 2007; Solomon et al. 2004) as well as some *Trachymyrmex* species (SES, unpublished). The nest entrance consists of a single hole surrounded by excavated soil (Figure 1A), and a single tunnel connects this entrance to the fungus chambers (Figure 1B). However, two deviations from the “typical” nest structure made these nests more difficult to excavate than those of most lower attines. First, instead of proceeding straight down from the surface, the tunnel near the nest entrance descended at an angle for several centimeters before making a sharp turn and descending downwards. Because the tunnel was narrow (3–4 mm in diameter) it could be easily lost, so care had to be taken to trace the tunnel for the first 10–20 centimeters beneath the entrance. Second, the most superficial garden-containing chamber was extremely deep. In the only nest in which any garden chambers were reached, the depth of the highest fungus chamber was 3.54 meters. In two of the other excavated nests, UGM080923-01 and TRS080923-01, the tunnel was traced to a depth of 2.16 meters and 2.90 meters, respectively, without encountering a fungus chamber. Excavations of the fourth nest, CTL080924-01, were abandoned because the tunnel was lost below the empty, superficial chamber and could not be relocated despite careful exploration to a depth of 1.65 meters.

Because the ants' tunnel was carefully traced during excavations, we are confident that no fungus chambers were missed in the parts of the nests that were excavated. It is likely that the reason a fungus chamber was not found in three of the four nests is that the chambers containing fungus gardens were still deeper. Two of the unsuccessful excavations (UGM080923-01 and TRS080923-01, which terminated at depths of 2.16 and 2.9 meters, respectively) found numerous chambers filled or partially filled with loose soil, suggesting that they had once been used as garden chambers, but had subsequently been abandoned.

It is possible that the ants relocate gardens seasonally, moving them to deeper chambers during the hot, dry season in which our excavations were conducted. Similar behavior has been documented for the North American leafcutter ants *Atta texana* and *Acromyrmex versicolor*, in which the fungus garden is spread out among many different chambers during summer months and then collapsed into relatively few deep chambers during the cooler winter (Moser 1962; Moser 2006). If *Mycetagroicus* fungus gardens are indeed kept below 3 meters during the driest and hottest months, the ants could be using the chambers closer to the surface as garden chambers during cooler/wetter months and as temporary dumps during the dry season. This would allow the ants to continue excavation without having to travel all the way to the surface to deposit the waste material, and would explain why debris was found in more superficial chambers. However, climate alone seems unlikely to be the sole factor determining the extreme garden depth in *M. cerradensis* because other Attini encountered at the same time at this site, including species of *Trachymyrmex, Sericomyrmex, Myrmicocrypta, Mycocepurus*, and *Cyphomyrmex* (of which the latter three presumably cultivate fungi closely related to that grown by *M. cerradensis*), had shallower nests of varying depth. Nest excavations of *M. cerradensis* at the same site during the wet season can address this paradox.

The fungus garden cultivated by *Mycetagroicus cerradensis* is closely related to fungi cultivated by other lower attines, especially *Cyphomyrmex* faunulus, *Mycocepurus smithii*, and *Myrmicocrypta* cf. *buenzlii* (Figure 2). The whitish nodules visible throughout the garden (Figure 1D) are not clumps of gongylidia (Figure 1F), as in higher attine fungi, but are clumps of normal hyphae (Figure 1E) that are also common in some lower attine cultivars (UGM and JSC, personal observations).

The phylogenetic position of *Mycetagroicus* ants as sister to *Trachymyrmexl Sericomyrmex* and the leafcutter ants (Schultz and Brady 2008), combined with the evidence shown here that the fungi cultivated by *Mycetagroicus* ants are in Clade 2 of the G3 fungi associated with most lower attines (Chapela et al. 1994; Mueller et al. 1998), suggest that the transition from lower to higher attine agriculture occurred in the common ancestor of *Trachymyrmex*/*Sericomyrmex* after the divergence of the *Mycetagroicus* lineage. This permits more accurate estimation of when this transition occurred, since the recent attempt to date the origin of higher attine agriculture (Schultz and Brady 2008) was adversely affected by the lack of information regarding the form of agriculture practiced by *Mycetagroicus*. Specifically, since it was not known which clade of fungi is cultivated by *Mycetagroicus* ants, the analysis by Schultz and Brady conservatively used the node that subtends the group (*Mycetagroicus* + higher attines) as the stem group for estimating a date for the origin of higher attine agriculture (see Figure 1 and Table S3 in Schultz and Brady 2008). Because it is now known that higher attine agriculture first evolved after the split between the ancestor of *Mycetagroicus* and the higher attines, the node that represents the common ancestor of *Mycetagroicus* and the higher attines can be used as the earliest possible date (i.e. the stem-group) for the origin of higher attine agriculture. Assuming a date of 73.5 mya for the root of the phylogenetic tree used to estimate divergence times (see Schultz and Brady 2008 for a complete discussion), the revised estimates for the date of this event (Table 1) range from 16–18 mya (for the crown-group and stem group, respectively) using a penalized likelihood model and 21–25 mya (for the crown-group and stem group, respectively) using a Bayesian uncorrelated lognormal approach. Because very little information is known about the basic biology and natural history of most species of *Trachymyrmex* and *Sericomyrmex*, especially those from South America, further insight into the details of this evolutionary transition must await additional data on these enigmatic taxa.

Although a total of 746 worker ants were collected from nest SES080924-05, this does not necessarily represent an accurate estimate of the colony size for this species for three reasons. First, although an attempt was made to collect every worker ant seen at the time of excavation, some individuals inevitably escaped or were away from the nest. Second, it is possible that additional tunnels or chambers were missed during excavation, although as indicated above no evidence was found for the existence of any such structures.

**Table 1.**
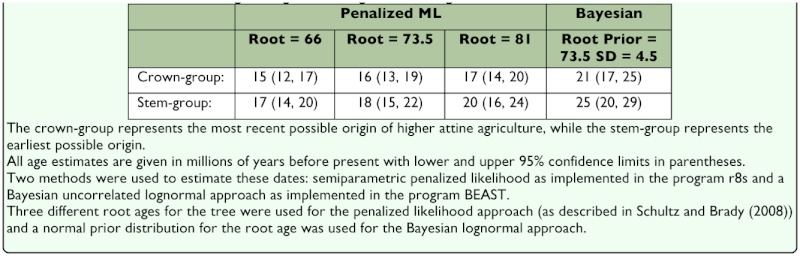
Estimated dates for the origin of higher attine agriculture using various methods.

Third, counts of the number of workers occurred after keeping fragments of the colony alive for several months, during which time additional workers eclosed that were immature brood at the time of excavation. Nevertheless, we estimate that the total colony size for this species is likely to be around 600 to 800 individuals, although future excavations will be needed to arrive at a more reliable estimate.

The presence of the fungal parasite *Escovopsis* was not detected in the one garden found. However, this single negative data point cannot be taken as an indication that *Escovopsis* does not infect *Mycetagroicus*, since the infection rate is known to be far less than 100% and to differ across localities (Currie et al. 1999; Rodrigues et al. 2008). However, *Escovopsis* was detected in nests of other attine species from the same site (ARS and SES, unpublished observations), suggesting that the potential exists for it to infect *Mycetagroicus* at this locality. Future research is required to confirm that *Escovopsis* does infect *Mycetagroicus* nests and to determine whether the specific strain is related to those that infect other lower attine species (Currie et al. 2003).

The survey of attine ants near Uberlândia, as well as previous surveys at the same site (Lopes and Vasconcelos 2008; Vasconcelos et al. 2008), suggest that *Mycetagroicus* *cerradensis* is relatively abundant in several types of *Cerrado* vegetation. This observation accords with the conclusion of Brandão and Mayhé-Nunes (2008) that *Mycetagroicus* species appear to be common, yet are not frequently collected. Further observations on the natural history of these species are needed to clarify their basic biology, such as their foraging behavior, nuptial flight activity, and ecological interactions with other species. However, the depth of their fungal chambers—at least in the dry season—makes it unlikely that *Mycetagroicus* will become a model system for South American attine ants.
